# Metformin, a Diabetes Drug, Eliminates Tumor-Initiating Hepatocellular Carcinoma Cells

**DOI:** 10.1371/journal.pone.0070010

**Published:** 2013-07-29

**Authors:** Tomoko Saito, Tetsuhiro Chiba, Kaori Yuki, Yoh Zen, Motohiko Oshima, Shuhei Koide, Tenyu Motoyama, Sadahisa Ogasawara, Eiichiro Suzuki, Yoshihiko Ooka, Akinobu Tawada, Motohisa Tada, Fumihiko Kanai, Yuichi Takiguchi, Atsushi Iwama, Osamu Yokosuka

**Affiliations:** 1 Department of Gastroenterology and Nephrology, Graduate School of Medicine, Chiba University, Chiba, Japan; 2 Department of Cellular and Molecular Medicine, Graduate School of Medicine, Chiba University, Chiba, Japan; 3 Institute of Liver Studies, King’s College Hospital, London, United Kingdom; 4 Department of Medical Oncology, Graduate School of Medicine, Chiba University, Chiba, Japan; University of Bari Medical School, Italy

## Abstract

Metformin has been widely used as an oral drug for diabetes mellitus for approximately 60 years. Interestingly, recent reports showed that metformin exhibited an anti-tumor action in a wide range of malignancies including hepatocellular carcinoma (HCC). In the present study, we investigated its impact on tumor-initiating HCC cells. Metformin suppressed cell growth and induced apoptosis in a dose-dependent manner. Flow cytometric analysis showed that metformin treatment markedly reduced the number of tumor-initiating epithelial cell adhesion molecule (EpCAM)^+^ HCC cells. Non-adherent sphere formation assays of EpCAM^+^ cells showed that metformin impaired not only their sphere-forming ability, but also their self-renewal capability. Consistent with this, immunostaining of spheres revealed that metformin significantly decreased the number of component cells positive for hepatic stem cell markers such as EpCAM and α-fetoprotein. In a xenograft transplantation model using non-obese diabetic/severe combined immunodeficient mice, metformin and/or sorafenib treatment suppressed the growth of tumors derived from transplanted HCC cells. Notably, the administration of metformin but not sorafenib decreased the number of EpCAM^+^ cells and impaired their self-renewal capability. As reported, metformin activated AMP-activated protein kinase (AMPK) through phosphorylation; however its inhibitory effect on the mammalian target of rapamycin (mTOR) pathway did not necessarily correlate with its anti-tumor activity toward EpCAM^+^ tumor-initiating HCC cells. These results indicate that metformin is a promising therapeutic agent for the elimination of tumor-initiating HCC cells and suggest as-yet-unknown functions other than its inhibitory effect on the AMPK/mTOR pathway.

## Introduction

Cancer stem cells (CSCs) or tumor-initiating cells (TICs) are a minor population of tumor cells with prominent tumorigenicity [1]. These cells are characterized by self-renewal capability and differentiation ability similar to those of normal stem/progenitor cells. Therefore, it has been believed that TICs play an important role in carcinogenesis, tumor growth, metastasis, and cancer recurrence. Recent progress in stem cell biology has enabled the identification and characterization of TICs in various cancers including hepatocellular carcinoma (HCC) [2]. Subsequently, the molecular machinery and signaling pathways involved in maintaining TICs have been vigorously explored [3]. Although the inhibitors of these molecules and signaling pathways are considered promising as TIC-targeting drugs, an effective therapy targeting TICs has yet to be developed.

Metformin is an oral drug that lowers blood glucose concentrations and has been widely used to treat type 2 diabetes mellitus [4]. The anti-diabetic action of metformin depends on the activation of AMP-activated protein kinase (AMPK), which contributes to a reduction in hepatic gluconeogenesis and an increase in glucose uptake in skeletal muscles [5]. Of interest, previous large case-control studies revealed that diabetic patients treated with metformin had a lower incidence of cancers than those treated with other diabetic drugs [6], [7]. Various explanations for the efficacy of metformin have been proposed, such as the activation of AMPK, inhibition of insulin-like growth factor signaling, and the mTOR pathway [8]. Diabetes is known to be associated with an increase in the risk of developing HCC [9]. Indeed, the risk of HCC was significantly lower with metformin treatment than with sulphonylureas or insulin in chronic liver disease [10]. Furthermore, metformin reduced the risk of recurrence of HCC after local ablation therapy [11]. Taken together, it is possible that metformin has direct effects on tumor-initiating HCC cells.

In the present study, we examined the effect of metformin on tumor-initiating HCC cells *in* *vitro*. We showed that metformin abolished their self-renewal capability and induced apoptosis in part through the activation of AMPK and subsequent inhibition of the mTOR pathway. Furthermore, xenograft transplantation experiments using nonobese diabetic/severe combined immunodeficient (NOD/SCID) mice demonstrated that metformin but not sorafenib decreased the number of TICs and impaired their self-renewal capability.

## Results

### Metformin Inhibited Cell Growth and Induced Apoptosis

**Figure 1:**
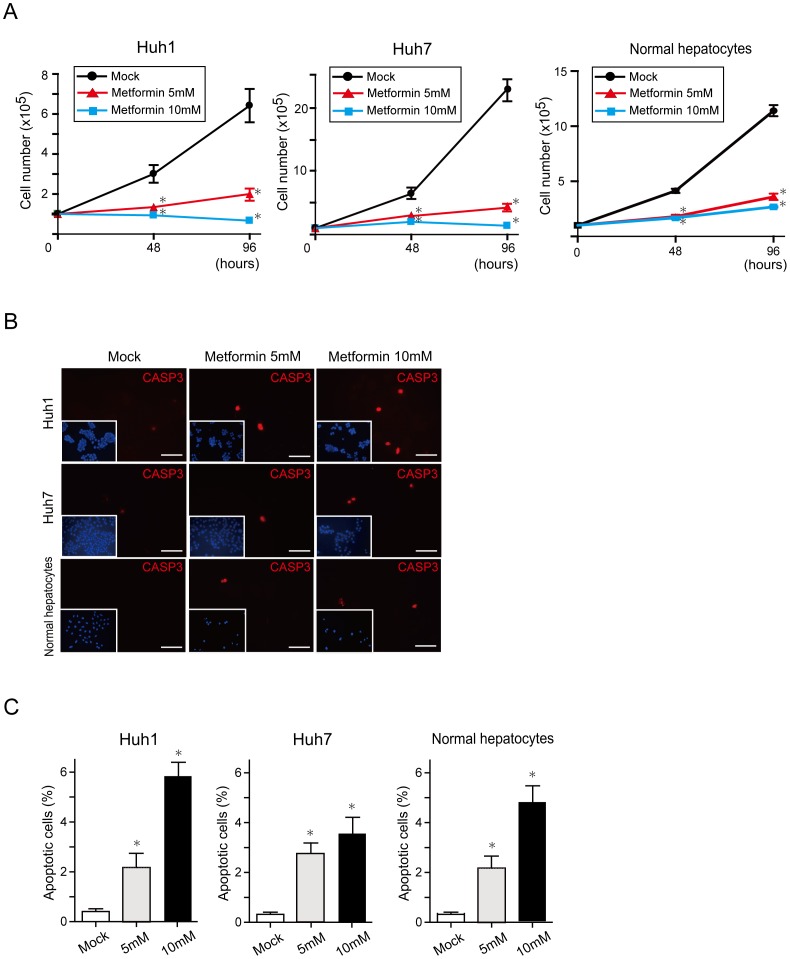
*In vitro* assays of HCC cells and normal hepatocytes treated with metformin. (A) Dose-dependent and time-dependent inhibition of the growth of HCC cells and normal hepatocytes treated with metformin. *Statistically significant (p<0.05). (B) Detection of apoptotic cells by immunostaining of CASP3. Nuclear DAPI staining is shown in the insets. Scale bar = 100 µm. (C) Quantification of the percentage of apoptotic cells. *Statistically significant (p<0.05).

**Figure 2:**
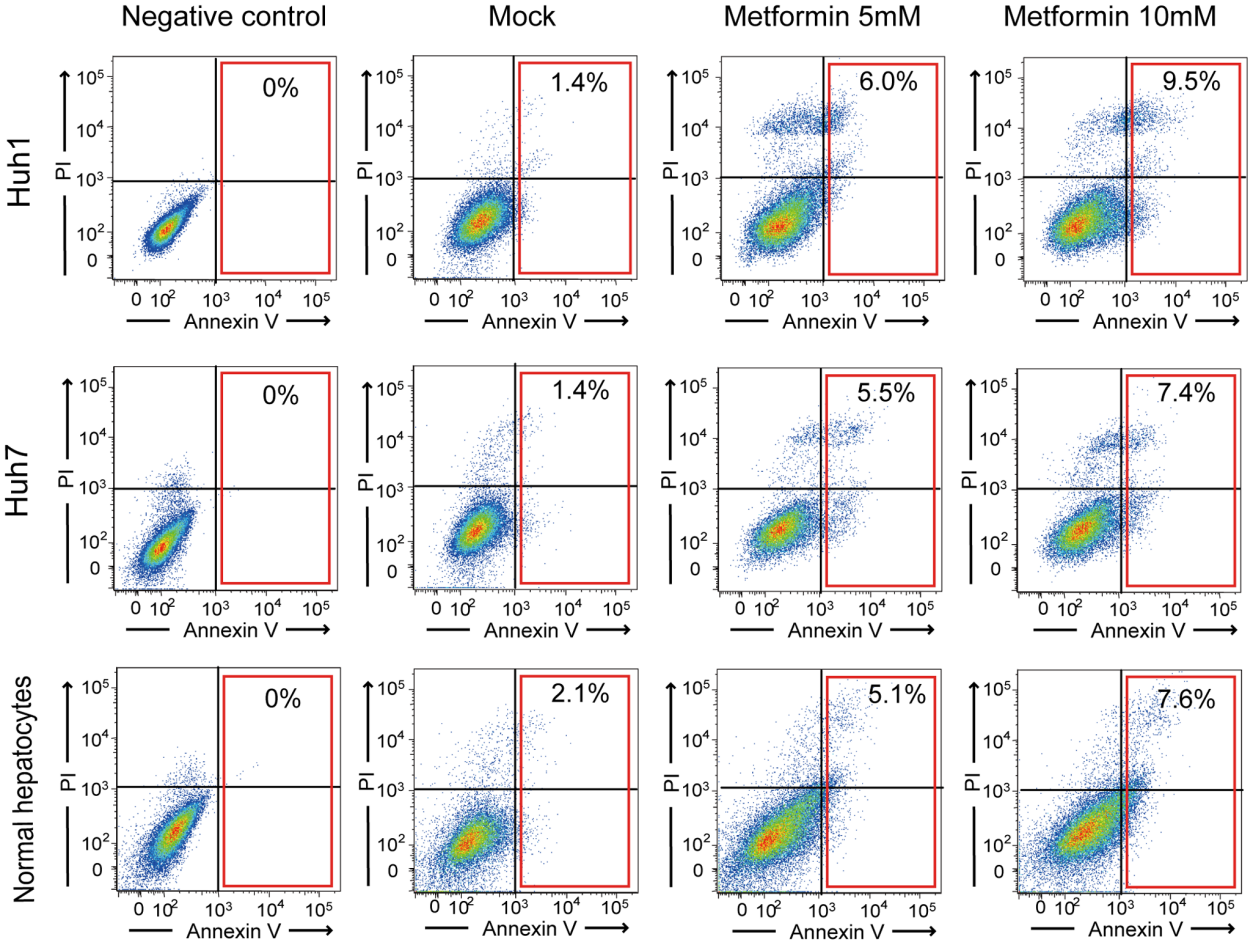
Detection of apoptotic cells by staining with Annexin V and PI using flow cytometry. The percentages of Annexin V-positive cells are shown as the mean values for three independent analyses.

To investigate the effect of metformin on HCC cells, we first examined cell growth and the frequency of apoptotic cells. Metformin treatment inhibited cell growth both time-dependently and dose-dependently in HCC cells and normal hepatocytes (Fig. 1A). Immunostaining of active caspase 3 (CASP3) showed that metformin induced apoptosis in a dose-dependent manner (Fig. 1B). The percentage of apoptotic cells in Huh1 cells, Huh7 cells, and normal hepatocytes treated with metformin (5 mM) was approximately five-fold, ten-fold, and seven-fold higher than that in control cells, respectively (Fig. 1C). Consistent with this result, flow cytometric analysis by staining with Annexin V and propidium iodide (PI) revealed that metformin caused apoptosis in a dose-dependent manner (Fig. 2).

### Impact of Metformin Treatment on Tumor-initiating HCC Cells

**Figure 3:**
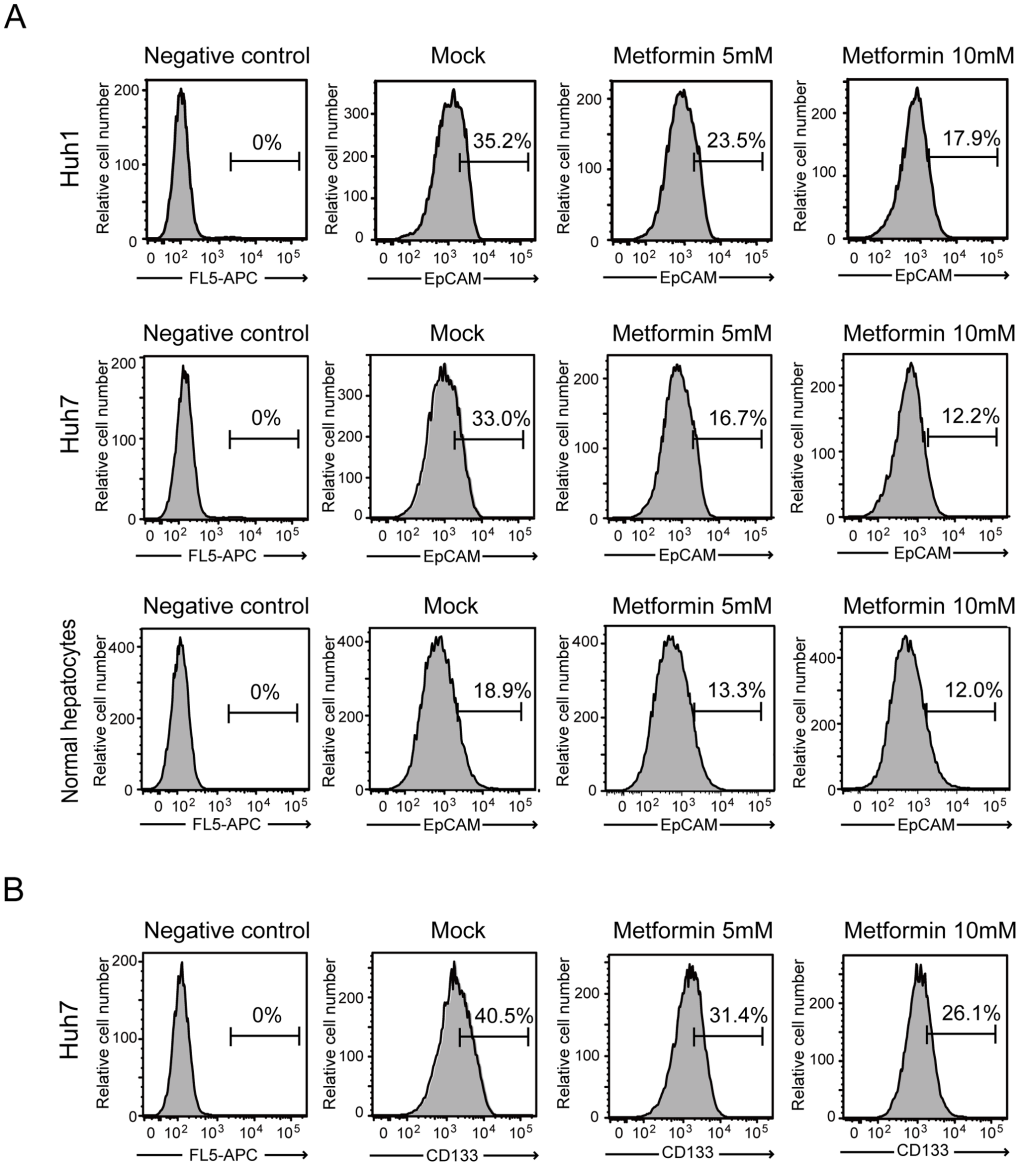
Flow cytometric profiles of HCC cells and normal hepatocytes treated with metformin (5 or 10 mM) for 72 hours. (A) The percentages of EpCAM^+^ fractions are shown as the mean values for three independent analyses. (B) The percentages of CD133^+^ fractions in Huh7 cells are shown as the mean values for three independent analyses.

The epithelial cell adhesion molecule (EpCAM)^+^ fraction as well as the CD133^+^ fraction was shown to include TICs in HCC [12], [13]. We examined the expression of EpCAM and CD133 using flow cytometry to analyze the effect of metformin on tumor-initiating HCC cells. Metformin treatment (10 mM) decreased the EpCAM^high^ fraction from 35.2% to 17.9% in Huh1 cells and from 33.0% to 12.2% in Huh7 cells (Fig. 3A). The EpCAM^high^ fraction also decreased from 18.9% to 12.0% in normal hepatocytes after metformin exposure (Fig. 3A). Likewise, the CD133^high^ fraction in Huh7 cells decreased from 40.5% to 26.1% (Fig. 3B), while the CD133^+^ fraction was not detected in Huh1 cells or normal hepatocytes with or without metformin treatment. Taking into consideration the decrease in the total cell number, metformin appears to directly act on tumor-initiating HCC cells.

### Sphere Assays of HCC Cells and Normal Hepatocytes Treated with Metformin

**Figure 4:**
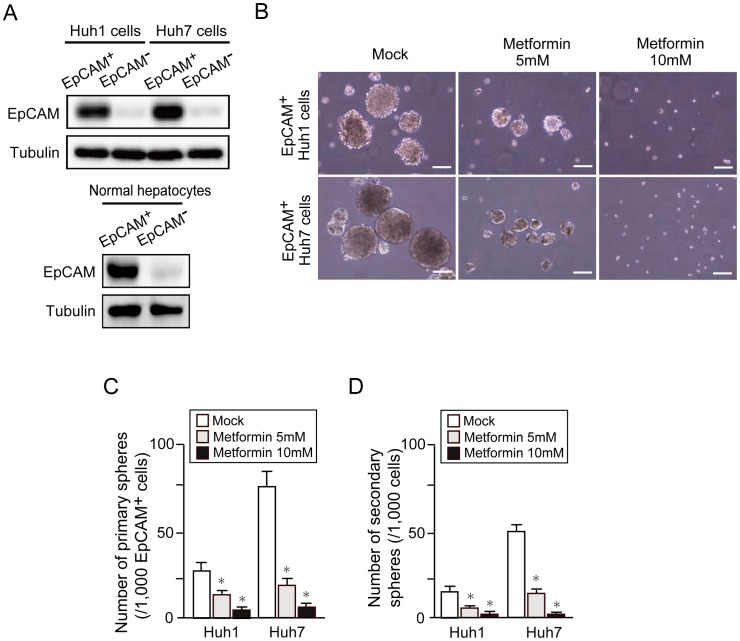
Non-adherent sphere formation assays of EpCAM^+^ cells treated with metformin. (A) Western blot analysis of EpCAM expression in sorted EpCAM^+^ cells. Tubulin was used as a loading control. (B) Bright-field images of the non-adherent spheres of EpCAM^+^ HCC cells at day 14 of culture. Scale bar = 100 µm. (C) Number of large spheres generated from 1,000 EpCAM^+^ cells treated with metformin. *Statistically significant (p<0.05). (D) Number of secondary spheres 14 days after replating. *Statistically significant (p<0.05).

**Figure 5:**
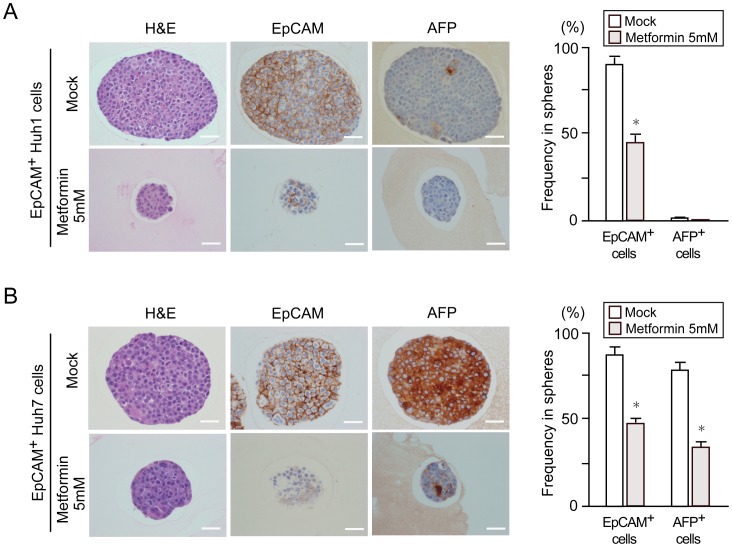
Immunostaining of EpCAM^+^ cell-derived spheres. (A) Hematoxylin and eosin staining and immunocytochemical analysis of EpCAM and AFP in spheres derived from EpCAM^+^ cells. Scale bar = 20 µm. (B) The percentage of EpCAM^+^ cells or AFP^+^ cells was determined. *, Statistically significant (p<0.05).

We then performed a non-adherent sphere formation assay of EpCAM^+^ HCC cells and normal hepatocytes sorted by flow cytometry. EpCAM expression was markedly higher in the EpCAM^+^ fraction than in the EpCAM^-^ fraction by Western blot analysis (Fig. 4A). Unlike EpCAM^+^ HCC cells, EpCAM^+^ normal hepatocytes failed to form large spheres. Metformin treatment significantly impaired the formation of large spheres dose-dependently (Fig. 4B and 4C) and also the formation of secondary spheres after the replating of primary spheres (Fig. 4D). Together, these results indicate that metformin impaired the tumorigenicity of tumor-initiating HCC cells by inhibiting their self-renewal. To confirm the inhibitory effect of metformin on the self-renewal of tumor-initiating HCC cells, we conducted immunocytochemical analyses of the expression of EpCAM and α-fetoprotein (AFP), hepatic stem/progenitor cell markers, in the resultant spheres. A marked reduction in cells positive for EpCAM was observed in spheres generated from EpCAM^+^ Huh1 cells (Fig. 5A). Because Huh1 cells only modestly produce AFP, no remarkable change in the number of cells positive for AFP was observed after metformin treatment. In contrast, metformin decreased both AFP^+^ and EpCAM^+^ cells in spheres generated from Huh7 cells treated with metformin (Fig. 5B). These results indicate that metformin impairs the self-renewal capability of TICs and accelerates their differentiation.

### Impact of Metformin on Apoptosis, Cell Growth, and Cell Cycle

**Figure 6:**
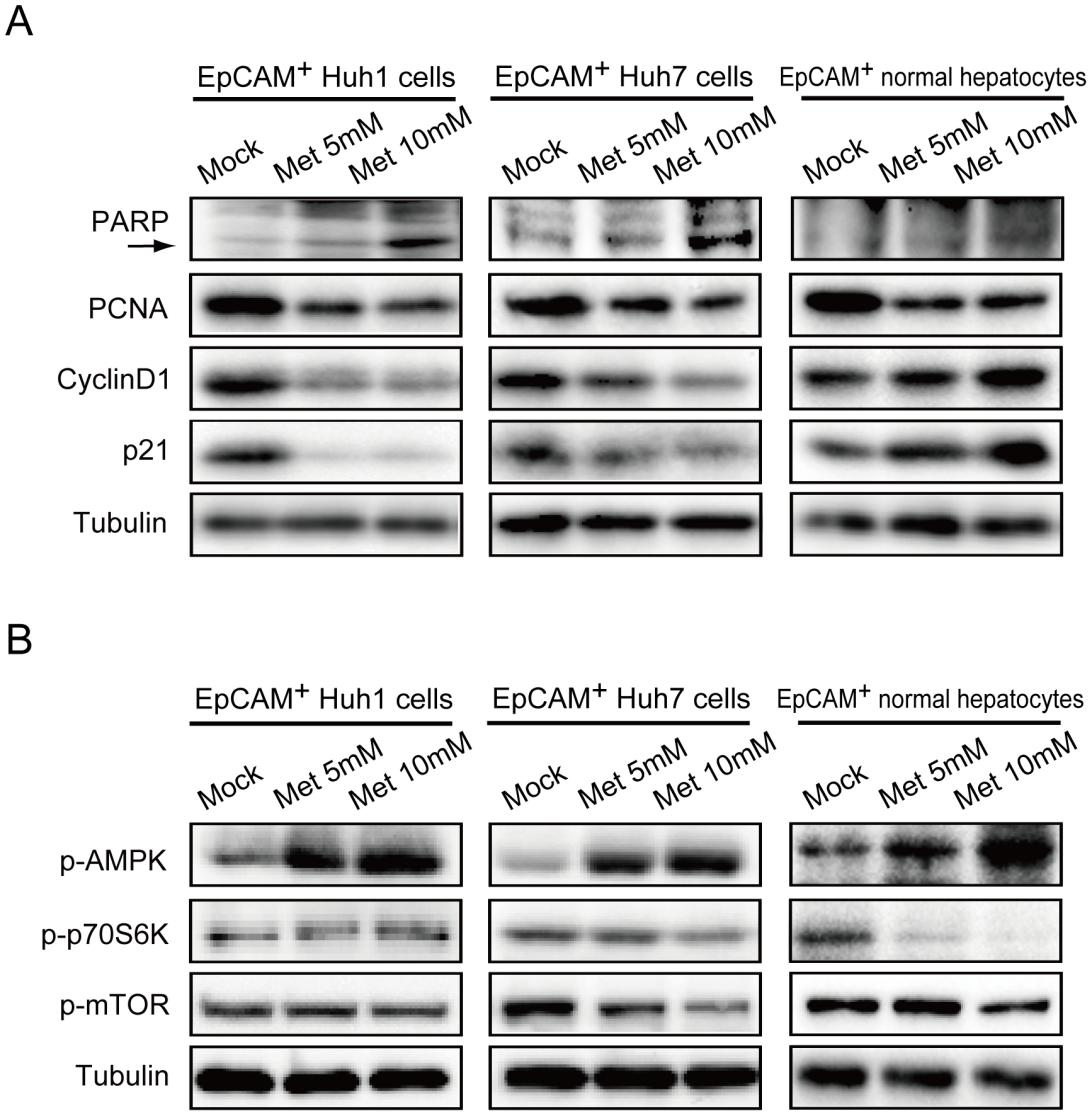
Cell growth inhibition, induction of apoptosis, and inhibition of the mTOR pathway after metformin treatment in HCC cells and normal hepatocytes. (A) EpCAM^+^ cells were subjected to Western blot analysis using anti-PARP, PCNA, cyclin D1, p21, and tubulin (loading control) antibodies. The arrow indicates the cleaved forms of PARP. (B) EpCAM^+^ cells were subjected to Western blotting using anti-phospho-AMPK, phospho-p70S6K, phospho-mTOR, and tubulin (loading control) antibodies.

To examine the effect of metformin on cell proliferation, we conducted Western blotting of EpCAM^+^ HCC cells and normal hepatocytes (Fig. 6A). As expected, cleaved poly (ADP-ribosyl) polymerase (PARP), a marker of apoptosis, was clearly detected in EpCAM^+^ cells treated with metformin. The levels of proliferating cell nuclear antigen (PCNA) in EpCAM^+^ cells treated with metformin decreased in a dose-dependent manner. The alteration in cyclin D1 and p21 expression levels caused by metformin differed between EpCAM^+^ HCC cells and EpCAM^+^ normal hepatocytes. Although metformin treatment increased the level of cyclin D1 in EpCAM^+^ HCC cells, no marked changes in cyclin D1 expression were observed in EpCAM^+^ normal hepatocytes. In addition, metformin treatment decreased the level of p21 in EpCAM^+^ HCC cells, but conversely increased p21 expression level in EpCAM^+^ normal hepatocytes.

### AMPK/mTOR Pathway Following Metformin Exposure in EpCAM^+^ HCC Cells and Normal Hepatocytes

Previous reports demonstrated that metformin suppressed mTOR signaling by activating AMPK in various cancer cells including HCC cells [14]–[16]. To examine whether this machinery also operated in EpCAM^+^ HCC cells and normal hepatocytes, cells were subjected to Western blotting (Fig. 6B). In EpCAM^+^ Huh7 cells and normal hepatocytes, the levels of phosphorylated AMPK increased after metformin exposure in a dose-dependent manner. Conversely, the levels of phosphorylated mTOR and phosphorylated S6 kinase decreased. However, EpCAM^+^ Huh1 cells showed no change in the mTOR pathway, while the level of AMPK increased with exposure to metformin. These results raise the possibility that metformin impaired the self-renewal capability of tumor-initiating HCC cells in part by affecting the AMPK/mTOR pathway.

### Anti-tumor Effects of Metformin and/or Sorafenib in Xenograft Transplantation Model

**Figure 7:**
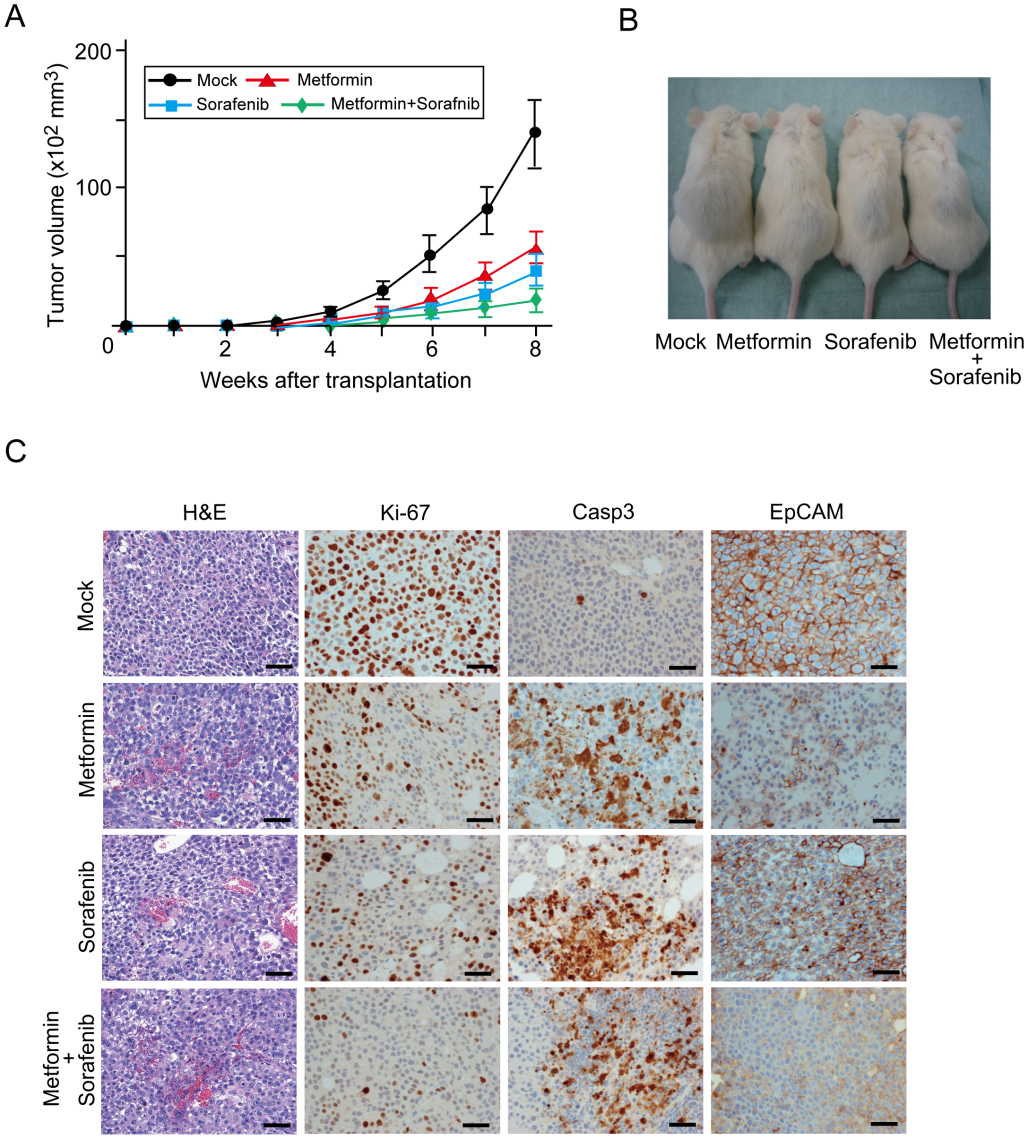
Inhibition of xenograft tumor growth by the administration of metformin and/or sorafenib. (A) A total of 2×10^6^ Huh7 cells were transplanted into NOD/SCID mice. Tumor volume was monitored weekly after the cell transplantation. *Statistically significant (p<0.05). (B) Representative images of recipient mice treated with metformin and/or sorafenib 6 weeks after the transplantation. (C) Hematoxylin and eosin (H&E) staining and immunohistochemical analysis of subcutaneous tumors.

Sorafenib is an oral multikinase inhibitor with anti-angiogenic activity and has been approved for the treatment of advanced HCC [17]. To compare the anti-tumor effects of metformin and sorafenib, we conducted xenograft transplantation using NOD/SCID mice and administered metformin and/or sorafenib to recipient mice. Metformin and/or sorafenib were administered daily after the transplantation of 2×10^6^ Huh7 cells into NOD/SCID mice. Both tumor initiation and growth were apparently suppressed by the metformin and sorafanib treatment (Fig. 7A and 7B). Tumor growth was further inhibited by the co-administration of metformin and sorafenib than by a single administration. Immunohistochemical staining of subcutaneous tumors for Ki-67 and CASP3 revealed that metformin and/or sorafanib treatment inhibited cell growth and induced apoptosis (Fig. 7C). Interestingly and importantly, the frequency of EpCAM^+^ cells was markedly decreased in tumors treated with metformin, but not with sorafenib (Fig. 7C). Co-treatment with metformin and sorafenib produced results similar to the single administration of metformin (Fig. 7C).

### Re-analysis of Subcutaneous Tumors

**Figure 8:**
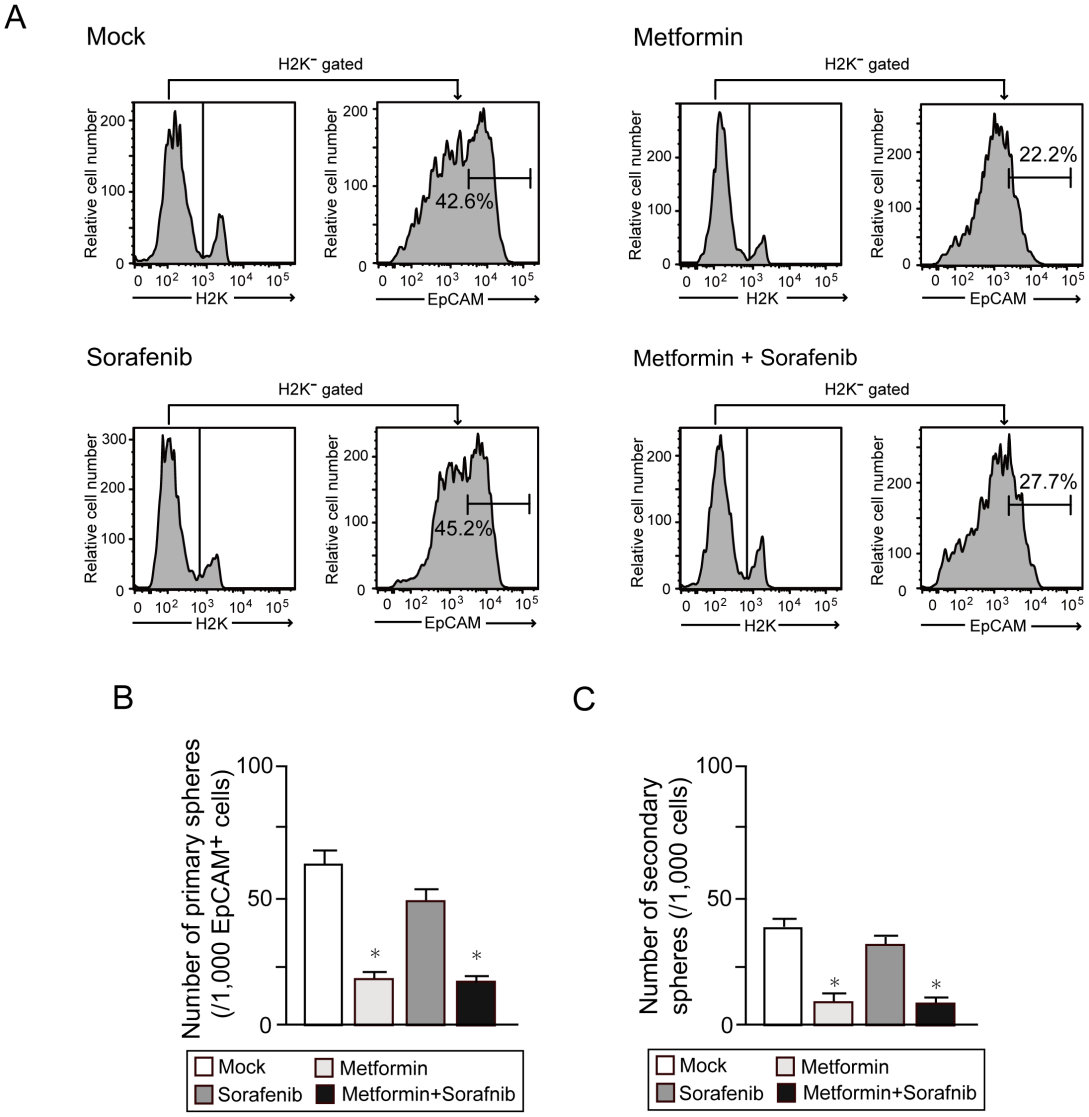
Re-analysis of xenograft tumors. (A) Flow cytometric analysis of subcutaneous tumors. The percentages of positive fractions for the indicated markers are shown as the mean values for three independent analyses. (B) Number of large spheres generated from 1,000 EpCAM^+^ HCC cells treated with metformin. *Statistically significant (p<0.05). (C) Number of secondary spheres 14 days after replating. *Statistically significant (p<0.05).

Consistent with the pathological findings, flow cytometric analysis of xenograft tumors clearly demonstrated that metformin markedly reduced the number of tumor-initiating EpCAM^+^ cells, whereas sorafenib did not (Fig. 8A). We conducted the non-adherent sphere formation assay of EpCAM^+^ cells isolated from subcutaneous tumors. Metformin treatment as well as co-treatment with sorafenib markedly impaired primary sphere formation and even more severely impaired secondary sphere formation (Fig. 8B and 8C). In contrast, sorafenib treatment had very little effect on the formation of primary and secondary spheres (Fig. 8B and 8C). Taken together, metformin could be a therapeutic agent for the elimination of tumor-initiating HCC cells, at least in part by inhibiting their self-renewal capacity.

## Discussion

A large number of studies have suggested that metformin has an anti-cancer effect in various types of malignancies including breast cancer and ovarian cancer, and even in HCC [14]–[16]. However, its efficacy against tumor-initiating HCC cells remains to be elucidated.

In this study, we first conducted cell growth assays in non-purified Huh1 and Huh7 cells treated with metformin. Consistent with previous reports, metformin treatment inhibited cell growth and induced apoptosis in both cell lines in dose-dependent and time-dependent manners. In addition, flow cytometric analysis showed a decrease in the proportion of EpCAM^+^ and CD133^+^ cells. These results prompted us to examine the direct action of metformin against tumor-initiating HCC cells. Sphere formation assays showed that metformin significantly suppressed the formation of spheres generated from EpCAM^+^ TICs in a dose-dependent manner. Subsequent analysis for secondary sphere formation after replating showed similar results to those of primary sphere assays. In addition, immunocytochemical analysis revealed that metformin treatment reduced the number of EpCAM^+^ and AFP^+^ cells in primary spheres. Taken together, it appears that metformin impaired EpCAM^+^ tumor-initiating HCC cells and simultaneously promoted the differentiation towards non-TICs.

Dependency on the mTOR pathway was shown to be higher in leukemic stem cells (LSCs) than in normal hematopoietic stem cells and the mTOR inhibitor rapamycin impaired the self-renewal of LSCs in leukemic mouse models [18]. mTOR signaling also makes a significant contribution to the maintenance of TICs in breast cancer and prostate cancer [19], [20]. The aberrant activation of mTOR signaling was also observed in approximately 50% of patients with HCC [21], [22]. The mTOR inhibitor, everolimus, which is currently undergoing clinical trials, exhibited an anti-tumor effect in some cases of advanced HCC [23]. Taken together, it appears that mTOR signaling plays an important role in hepatocarcinogenesis and the progression of HCC.

In the present study, metformin treatment apparently inhibited the mTOR pathway by phosphorylating AMPK in EpCAM^+^ Huh7 cells. Considering that mTOR inhibitors suppressed the growth of Huh7 cells not only in culture but also in xenograft models [24], [25], it is assumed that metformin exerted its anti-TIC effect by affecting the AMPK/mTOR pathway. In contrast, metformin did not alter the activity of the mTOR pathway in EpCAM^+^ Huh1 cells despite inducing the phosphorylation of AMPK. One possible explanation for this is that mutations in the components of the mTOR pathway, which have been identified in many cancers and cancer cell lines, mask the effect of metformin in EpCAM^+^ Huh1 cells [26]. Another possibility is that the mTOR pathway is not the major target of metformin and anti-tumor activity is exerted independent of the pathway. Several reports support this notion. For example, metformin was shown to cause cell cycle arrest by downregulating the expression of cyclin D1 and/or upregulating that of cyclin-dependent kinase inhibitors such as p21^Cip1^ without inhibiting the mTOR pathway [27], [28]. A previous report also attributed the anti-tumor activity of metformin to NF-kB inhibition in breast CSCs [29]. In addition, a recent study revealed a novel mechanism whereby metformin blocked glucagon-dependent glucose output from hepatocytes by reducing cyclic AMP and protein kinase A levels [30]. In the present study, metformin treatment suppressed the expression of cyclin D1 in EpCAM^+^ HCC cells, but not in EpCAM^+^ normal hepatcytes. Conversely, metformin increased p21 expression in EpCAM^+^ normal hepatcytes, but not in EpCAM^+^ HCC cells. Further analyses on the mechanisms of the anti-TIC activity of metformin are required.

Sorafenib is the sole molecular target drug clinically approved to treat advanced HCC. However, phase III trials have shown that sorafenib prolonged median overall survival of patients with advanced HCC by no more than 3 months [31], [32]. In our xenograft transplantation assay, treatment with metformin and sorafenib similarly suppressed the growth of subcutaneous tumors and co-treatment appeared to be more effective. Interestingly, both flow cytometric analysis and immunohistochemical analysis of xenograft tumors revealed that metformin significantly reduced the number of tumor-initiating EpCAM^+^ cells, whereas sorafenib treatment had minimal effects on TICs. Taking these results into consideration, it is possible that the combined use of metformin and sorafenib exhibited stronger anti-tumor effect than sorafenib treatment alone in HCC.

In summary, metformin reduced the number and tumorigenicity of tumor-initiating HCC cells; however the involvement of the AMPK/mTOR pathway in its anti-tumor activity remains ambiguous. Because metformin suppressed cell growth and decreased the number of EpCAM^+^ normal hepatocytes, further analysis might be necessary to determine whether metformin affects the function of normal hepatic stem/progenitor cells [33]. It is of importance to examine whether metformin might be of use for the elimination of TICs in HCC in clinical trials.

## Materials and Methods

### Ethics Statement

All experiments using mice were performed in accordance with our institutional guidelines for the use of laboratory animals and approved by the Review Board for Animal Experiments of Chiba University (approval ID: 22–187).

### Mice and Reagents

NOD/SCID mice (Sankyo Laboratory Co. Ltd., Tsukuba, Japan) were bred and maintained in accordance with our institutional guidelines for the use of laboratory animals (approval ID: 22–187). Metformin (1,1-dimethylbiguanide hydrochloride) and sorafenib tosylate were purchased from Sigma-Aldrich (St. Louis, MO) and LKT laboratories (Saint Paul, MN), respectively.

### Cell Culture and Sphere Formation Assay

The HCC cell lines, Huh1 and Huh7, were obtained from the Health Science Research Resources Bank (HSRRB, Osaka, Japan). Normal human hepatocytes were obtained from ACBRI (Kirkland, WA, USA). Cells were cultured in Dulbecco’s modified Eagle’s medium (Invitrogen Life Technologies, Carlsbad, CA) containing 10% fetal calf serum and 1% penicillin/streptomycin (Invitrogen). One thousand cells were plated onto ultra-low attachment six-well plates (Corning, Corning, NY) for the sphere formation assay. The number of spheres (>100 µm in diameter) was counted on day 14 of culture. A single cell suspension derived from original spheres was obtained for the secondary sphere formation using a Neurocult chemical dissociation kit (StemCell Technologies, Vancouver, BC, Canada). Paraffin-embedded sections of spheres were subjected to hematoxylin & eosin (H&E) staining and immunostaining with anti-EpCAM (Cell Signaling Technology, Danvers, MA) and anti-AFP (Dako Cytomation, Carpinteria, CA) antibodies for the pathological analysis.

### Growth Curves

The proliferation of HCC cells treated with metformin was examined using trypan blue staining after 48 and 96 hours of culture.

### Detection of Apoptotic Cells

To detect apoptosis, cells were stained with an anti-CASP3 antibody (Chemicon, Temecula, CA), followed by Alexa-555-conjugated goat anti-rabbit IgG (Molecular Probes). Apoptotic cells were also evaluated by staining with Annexin V-allophycocyanin (APC) (BD Biosciences, San Jose, CA) and PI using FACSCanto (BD Biosciences).

### Cell Sorting and Analysis

Single-cell suspensions were stained with an APC-conjugated anti-EpCAM antibody (Biolegend, San Diego, CA) or APC-conjugated anti-CD133/1 antibody (Miltenyi Biotec, Auburn, CA). After incubation, 1 µg/ml of PI was added to eliminate dead cells. Flow cytometric cell sorting and analysis were performed using FACSAria or FACSCanto (BD Biosciences).

### Western Blotting

Sorted HCC cells were subjected to Western blot analysis using anti-EpCAM (Abcam, Canbridge, UK) and anti-tubulin (Oncogene Science, Cambridge, MA) antibodies. Metformin-treated cells were also subjected to Western blotting using anti-PARP (Cell Signaling Technology), anti-PCNA (Santa Cruz Biotechnologies, Santa Cruz, CA), anti-cyclin D1 (BD Biosciences), anti-p21 (Cell Signaling Technology), anti-phospho-AMPK (Cell Signaling Technology), anti-phospho-mTOR (Ser2448, Cell Signaling Technology), anti-phospho-p70 S6 Kinase (Thr389, Cell Signaling Technology), and anti-tubulin antibodies.

### Xenograft Transplantation Using NOD/SCID Mice

In the metformin and/or sorafenib treatment model, a total of 2×10^6^ Huh7 cells were transplanted into the subcutaneous space of the backs of NOD/SCID mice. Metformin (250 mg/Kg, by intraperitoneal injection) and sorafenib (10 mg/Kg, by gavage) were administered daily. Tumor formation and growth were observed weekly. To analyze subcutaneous tumors, small pieces of tumors were put in DMEM containing 5 mg/ml collagenase type II (Roche) and digested. The cell suspension was centrifuged on Ficoll (IBL, Gunma, Japan) to remove dead cells and debris. Harvested cells were subjected to flow cytometric analyses and sphere formation assays. Subcutaneous tumors were also subjected to H&E staining and immunohistochemical staining with an anti-EpCAM antibody (Cell Signaling Technology), anti-CASP3 antibody (Chemicon), and anti-Ki67 antibody (DAKO, Carpinteria, CA). These experiments were performed in accordance with the institutional guidelines for the use of laboratory animals.

### Statistical Analysis

Data are presented as the mean ± SEM. Significant differences between 2 groups were analyzed using the Mann-Whitney U test. P values less than 0.05 were considered significant.

## References

[1] Jordan CT, Guzman ML, Noble M (2006) Cancer stem cells. N Engl J Med 355: 1253–1261. 16990388 10.1056/NEJMra061808

[2] Ji J, Wang XW (2012) Clinical implications of cancer stem cell biology in hepatocellular carcinoma. Semin Oncol 39: 461–472. 22846863 10.1053/j.seminoncol.2012.05.011 PMC3409471

[3] Visvader JE, Lindeman GJ (2008) Cancer stem cells in solid tumours: accumulating evidence and unresolved questions. Nat Rev Cancer 8: 755–768. 18784658 10.1038/nrc2499

[4] Bailey CJ, Turner RC (1996) Metformin. N Engl J Med 334: 574–579. 8569826 10.1056/NEJM199602293340906

[5] Zhou G, Myers R, Li Y, Chen Y, Shen X, et al (2001) Role of AMP-activated protein kinase in mechanism of metformin action. J Clin Invest 108: 1167–1184. 11602624 10.1172/JCI13505 PMC209533

[6] Evans JM, Donnelly LA, Emslie-Smith AM, Alessi DR, Morris AD (2005) Metformin and reduced risk of cancer in diabetic patients. BMJ 330: 1304–1305. 15849206 10.1136/bmj.38415.708634.F7 PMC558205

[7] Bowker SL, Majumdar SR, Veugelers P, Johnson JA (2006) Increased cancer-related mortality for patients with type 2 diabetes who use sulfonylureas or insulin. Diabetes Care 29: 254–258. 16443869 10.2337/diacare.29.02.06.dc05-1558

[8] Jalving M, Gietema JA, Lefrandt JD, de Jong S, Reyners AK, et al (2010) Metformin: taking away the candy for cancer? Eur J Cancer 46: 2369–2380. 20656475 10.1016/j.ejca.2010.06.012

[9] Davila JA, Morgan RO, Shaib Y, McGlynn KA, El-Serag HB (2005) Diabetes increases the risk of hepatocellular carcinoma in the United States: a population based case control study. Gut 54: 533–539. 15753540 10.1136/gut.2004.052167 PMC1774454

[10] Donadon V, Balbi M, Mas MD, Casarin P, Zanette G (2010) Metformin and reduced risk of hepatocellular carcinoma in diabetic patients with chronic liver disease. Liver Int 30: 750–758. 20331505 10.1111/j.1478-3231.2010.02223.x

[11] Chen TM, Lin CC, Huang PT, Wen CF (2011) Metformin associated with lower mortality in diabetic patients with early stage hepatocellular carcinoma after radiofrequency ablation. J Gastroenterol Hepatol 26: 858–865. 21251068 10.1111/j.1440-1746.2011.06664.x

[12] Yamashita T, Ji J, Budhu A, Forgues M, Yang W, et al (2009) EpCAM-positive hepatocellular carcinoma cells are tumor-initiating cells with stem/progenitor cell features. Gastroenterology 136: 1012–1024. 19150350 10.1053/j.gastro.2008.12.004 PMC2828822

[13] Ma S, Chan KW, Hu L, Lee TK, Wo JY, et al (2007) Identification and characterization of tumorigenic liver cancer stem/progenitor cells. Gastroenterology 132: 2542–2556. 17570225 10.1053/j.gastro.2007.04.025

[14] Qu Z, Zhang Y, Liao M, Chen Y, Zhao J, et al (2012) In vitro and in vivo antitumoral action of metformin on hepatocellular carcinoma. Hepatol Res 42: 922–933. 22524458 10.1111/j.1872-034X.2012.01007.x

[15] Shank JJ, Yang K, Ghannam J, Cabrera L, Johnston CJ, et al (2012) Metformin targets ovarian cancer stem cells in vitro and in vivo. Gynecol Oncol 127: 390–397. 22864111 10.1016/j.ygyno.2012.07.115 PMC3580263

[16] Hirsch HA, Iliopoulos D, Tsichlis PN, Struhl K (2009) Metformin selectively targets cancer stem cells, and acts together with chemotherapy to block tumor growth and prolong remission. Cancer Res 69: 7507–7511. 19752085 10.1158/0008-5472.CAN-09-2994 PMC2756324

[17] Gollob JA, Wilhelm S, Carter C, Kelley SL (2006) Role of Raf kinase in cancer: therapeutic potential of targeting the Raf/MEK/ERK signal transduction pathway. Semin Oncol 33: 392–406. 16890795 10.1053/j.seminoncol.2006.04.002

[18] Yilmaz OH, Valdez R, Theisen BK, Guo W, Ferguson DO, et al (2006) Pten dependence distinguishes haematopoietic stem cells from leukaemia-initiating cells. Nature 441: 475–482. 16598206 10.1038/nature04703

[19] Zhou J, Wulfkuhle J, Zhang H, Gu P, Yang Y, et al (2007) Activation of the PTEN/mTOR/STAT3 pathway in breast cancer stem-like cells is required for viability and maintenance. Proc Natl Acad Sci U S A 104: 16158–16163. 17911267 10.1073/pnas.0702596104 PMC2042178

[20] Dubrovska A, Kim S, Salamone RJ, Walker JR, Maira SM, et al (2009) The role of PTEN/Akt/PI3K signaling in the maintenance and viability of prostate cancer stem-like cell populations. Proc Natl Acad Sci U S A 106: 268–273. 19116269 10.1073/pnas.0810956106 PMC2629188

[21] Villanueva A, Chiang DY, Newell P, Peix J, Thung S, et al (2008) Pivotal role of mTOR signaling in hepatocellular carcinoma. Gastroenterology 135: 1972–1983. 18929564 10.1053/j.gastro.2008.08.008 PMC2678688

[22] Bhat M, Sonenberg N, Gores G (2013) The mTOR pathway in hepatic malignancies. Hepatology in press. 10.1002/hep.26323 PMC3688698 23408390

[23] Zhu AX, Abrams TA, Miksad R, Blaszkowsky LS, Meyerhardt JA, et al (2011) Phase 1/2 study of everolimus in advanced hepatocellular carcinoma. Cancer 117: 5094–5102. 21538343 10.1002/cncr.26165 PMC3417818

[24] Shirouzu Y, Ryschich E, Salnikova O, Kerkadze V, Schmidt J, et al (2010) Rapamycin inhibits proliferation and migration of hepatoma cells in vitro. J Surg Res 159: 705–713. 19482307 10.1016/j.jss.2008.07.035

[25] Villanueva A, Chiang DY, Newell P, Peix J, Thung S, et al (2008) Pivotal role of mTOR signaling in hepatocellular carcinoma. Gastroenterology 135: 1972–1983. 18929564 10.1053/j.gastro.2008.08.008 PMC2678688

[26] Huang S, Bjornsti MA, Houghton PJ (2003) Rapamycins: mechanism of action and cellular resistance. Cancer Biol Ther 2: 222–232. 12878853 10.4161/cbt.2.3.360

[27] Zhuang Y, Miskimins WK (2008) Cell cycle arrest in Metformin treated breast cancer cells involves activation of AMPK, downregulation of cyclin D1, and requires p27Kip1 or p21Cip1. J Mol Signal 3: 18. 19046439 10.1186/1750-2187-3-18 PMC2613390

[28] Chen HP, Shieh JJ, Chang CC, Chen TT, Lin JT, et al (2013) Metformin decreases hepatocellular carcinoma risk in a dose-dependent manner: population-based and in vitro studies. Gut 62: 606–615. 22773548 10.1136/gutjnl-2011-301708

[29] Hirsch HA, Iliopoulos D, Struhl K (2013) Metformin inhibits the inflammatory response associated with cellular transformation and cancer stem cell growth. Proc Natl Acad Sci U S A 110: 972–977. 23277563 10.1073/pnas.1221055110 PMC3549132

[30] Miller RA, Chu Q, Xie J, Foretz, Viollet B, et al (2013) Biguanides suppress hepatic glucagon signalling by decreasing production of cyclic AMP. Nature 494: 256–260. 23292513 10.1038/nature11808 PMC3573218

[31] Cheng AL, Kang YK, Chen Z, Tsao CJ, Qin S, et al (2009) Efficacy and safety of sorafenib in patients in the Asia-Pacific region with advanced hepatocellular carcinoma: a phase III randomised, double-blind, placebo-controlled trial. Lancet Oncol 10: 25–34. 19095497 10.1016/S1470-2045(08)70285-7

[32] Llovet JM, Ricci S, Mazzaferro V, Hilgard P, Gane E, et al (2008) SHARP Investigators Study Group, Sorafenib in advanced hepatocellular carcinoma. N Engl J Med 359: 378–390. 18650514 10.1056/NEJMoa0708857

[33] Schmelzer E, Zhang L, Bruce A, Wauthier E, Ludlow J, et al (2007) Human hepatic stem cells from fetal and postnatal donors. J Exp Med 204: 1973–1987. 17664288 10.1084/jem.20061603 PMC2118675

